# Investigation of the Potential Association Between Atherosclerotic Cardiovascular Disease Risk Score and Diabetic Retinopathy in Patients with Type 2 Diabetes: A Cross-Sectional Study

**DOI:** 10.3390/biomedicines13030633

**Published:** 2025-03-05

**Authors:** Chrysa Agapitou, Theodoros N. Sergentanis, Effie G. Papageorgiou, Panagiotis Theodossiadis, Ignatios Ikonomidis, Vaia Lambadiari, Irini Chatziralli

**Affiliations:** 1 2nd Department of Ophthalmology, Attikon University Hospital, Medical School, National and Kapodistrian University of Athens, 12462 Athens, Greece; 2 Department of Public Health Policy, School of Public Health, University of West Attica, 12243 Athens, Greece; 3 Department of Biomedical Sciences, University of West Attica, 12243 Athens, Greece; 4 2nd Department of Cardiology, Attikon University Hospital, Medical School, National and Kapodistrian University of Athens, 12462 Athens, Greece; 5 2nd Department of Internal Medicine, Research Institute and Diabetes Center, National and Kapodistrian University of Athens, 12462 Athens, Greece

**Keywords:** diabetic retinopathy, cardiovascular risk, diabetes, retina, prevention

## Abstract

**Purpose**: To examine the association between diabetic retinopathy (DR) and the atherosclerotic cardiovascular disease (ASCVD) risk score using the “ASCVD Risk Estimator Plus” tool in patients with type 2 diabetes mellitus (DM) and to assess risk factors potentially associated with DR. **Methods**: Participants in the study included 181 patients with type 2 DM who underwent a thorough ophthalmic examination, including a best-corrected visual acuity (BCVA) measurement, a dilated fundoscopy, fundus photography, an optical coherence tomography (OCT), and an OCT-angiography (OCT-A). DR was graded as no apparent retinopathy (NDR), mild non-proliferative (NPDR), moderate NPDR, severe NPDR, or proliferative DR (PDR). In addition, a detailed medical history of patients was recorded, while the “ASCVD Risk Estimator Plus” tool by the American College of Cardiology was used to calculate the ASCVD risk. **Results**: The ASCVD score, derived by the “ASCVD Risk Estimator Plus”, was not found to be significantly correlated with DR (*p* = 0.191). Multivariable logistic regression analysis showed that factors associated with DR independently included DM duration (multivariable OR = 3.16, 95% CI: 1.55–6.44, *p* = 0.002), HbA1c levels (multivariable OR = 2.94, 95% CI: 1.37–6.32, *p* = 0.006), and the presence of neuropathy (multivariable OR = 3.59, 95% CI: 1.43–9.05, *p* = 0.007). In the multivariable multinomial logistic regression analysis, NPDR development was associated with duration of DM (multivariable RR = 3.31, 95% CI: 1.57–6.97, *p* = 0.002), HbA1c levels (multivariable RR = 2.24, 95% CI: 1.00–5.02, *p* = 0.050), and neuropathy (multivariable RR: 3.94, 95% CI: 1.54–10.11, *p* = 0.004), while PDR development was only associated with HbA1c levels (multivariable RR = 6.88, 95% CI: 2.19–21.63, *p* = 0.001). **Conclusions**: The ASCVD score, as it was calculated using the “ASCVD Risk Estimator Plus” tool, was not found to be significantly associated with DR. Factors significantly associated with DR were DM duration, HbA1c levels, and the presence of neuropathy.

## 1. Introduction

Diabetes mellitus (DM) is a global epidemic, which is estimated to affect about 785 million people worldwide in 2045 [1]. Diabetic retinopathy (DR) is a microvascular complication of DM and a leading cause of visual impairment, especially in the working-age population [2,3]. On the other hand, atherosclerotic cardiovascular disease (ASCVD), defined as coronary heart disease, cerebrovascular disease, or peripheral arterial disease presumed to be of atherosclerotic origin, is the leading cause of morbidity and mortality for individuals with DM [4]. Specifically, patients with DM type 2 have a 2- to 4-fold increase in risk of incident coronary heart disease and ischemic stroke, as well as a 1.5 to 3.6-fold increase in mortality [5]. Taking into account the increasing number of cardiovascular event survivors and the global epidemic of DM, it is expected that a rise in the number of patients with type 2 DM at a higher cardiovascular disease (CVD) risk will occur, posing a giant challenge for health care systems worldwide, while cost-effective policies for reducing CVD risk in this population remain an unmet need [6]. It is worth noting that DR has been associated with an increased CVD incidence, independent of other known cardiovascular risk factors [7]. In fact, DR is an indication of active and uncontrolled DM and thereby increases the risk of CVD [8]. In addition, higher degrees of DR appear to have an increased risk for CVD and all-cause mortality in patients with type 2 DM [9,10,11], also pointing out that proliferative DR (PDR) can be a predictor of coronary heart disease [11,12,13]. Although the value of DR in predicting cardiovascular events is considered to be low [14], a recent study by Kha et al. showed that in individuals with high CVD risk, the presence of DR independently predicts increased CVD mortality, underlining the additional contribution of microvascular disease to CVD mortality [15].

A number of clinical risk tools are recommended to help estimate ASCVD risk in clinical practice, although several concerns have been raised about the use of widely used scores in patients with type 2 DM [16,17]. A recent study in China used the China-PAR equations to estimate the 10-year ASCVD risk among individuals free of ASCVD and found that the 10-year ASCVD risk was not predictive of DR [18]. The American College of Cardiology/American Heart Association “ASCVD Risk Estimator Plus” is an online tool, which is used to estimate the 10-year risk of a first ASCVD event (non-fatal myocardial infarction or coronary heart disease death, or fatal/non-fatal stroke) by entering definite risk factors and to forecast the potential impact of different interventions on patient risk [4,19]. This tool considers demographic data (age, sex, and race), smoking habits, blood pressure data, cholesterol, LDL and HDL values, and a history of DM, as well as hypertension treatment, while the 10-year risk is based on the first ASCVD event over a 10-year period among people previously free from ASCVD [19].

Based on the above, the purpose of this study was to examine the association between DR and the ASCVD risk score in patients with type 2 DM, using the “ASCVD Risk Estimator Plus” tool, since we hypothesize that DR may be a predictor of ASCVD risk. Additionally, we assessed risk factors potentially associated with DR.

## 2. Materials and Methods

This cross-sectional study included 181 patients with type 2 DM, who were recruited at the 2nd Department of Ophthalmology, National and Kapodistrian University of Athens, Athens, Greece, between 1 April 2023 and 31 December 2023. Patients with other retinal diseases than DR, as well as those with uncontrolled glaucoma (intraocular pressure > 30 mmHg), uveitis, and cornea or lens opacities, were excluded from the study. In addition, after initial recruitment, patients who had previous cardiovascular events, namely heart attack and stroke, were not included in the analysis. The study was conducted in accordance with the Helsinki Declaration, and written informed consent was obtained by all participants before enrollment in the study. The study was approved by the Institutional Review Board of Attikon University Hospital (Ref: 302/2021).

We recorded demographic data (age and sex), social habits (smoking), and thorough medical history, including comorbidities (hypertension and dyslipidemia), medications used, DM duration, history of stroke or heart attack, and the presence of DM complications (DR, neuropathy, nephropathy, and diabetic foot). Systolic (SBP) and diastolic blood pressure (DBP) were measured in all participants twice, and we calculated the mean of the measurements for statistical analysis. Moreover, all patients underwent a forearm venous puncture for peripheral blood extraction, and serum was separated. Biochemical analyses were performed on a Roche Cobas 8000 (Roche, Chicago, IL, USA) in the laboratory of Attikon University Hospital. Specifically, we analyzed the following parameters: glucose, glycated Hb (HbA1c), cholesterol, HDL, LDL, and triglycerides.

Additionally, the “ASCVD 10-Year Risk estimator plus” was used to calculate the 10-year risk of a first ASCVD [19]. Patients over 79 years were excluded since the tool is suitable for individuals 40–79 years old.

Regarding ophthalmologic examination, all participants underwent best-corrected visual acuity (BCVA) measurement, slit-lamp examination, dilated fundoscopy, and ocular imaging, including color fundus photographs, optical coherence tomography (OCT), and OCT-angiography (OCT-A) using DRI Triton (Topcon), while fundus fluorescein angiography (FFA) using Spectralis (Spectralis HRA + OCT, Heidelberg Engineering, Germany) was performed at the physician’s discretion to rule out the presence of neovascularization.

Diabetic retinopathy was classified according to the International Clinical Diabetic Retinopathy Disease Severity Scale as no DR, mild non-proliferative DR (NPDR), moderate NPDR, severe NPDR, and proliferative DR (PDR). The presence of diabetic macular edema (DME) was also evaluated.

The primary objective was to investigate any significant associations between ASCVD risk, as it was calculated by the ASCVD estimator plus tool, and DR or DME. In addition, other demographic and clinical characteristics of patients were examined as potential factors associated with DR development.

### Statistical Analysis

For the description of patients’ characteristics, descriptive statistics were calculated; mean and standard deviation (SD) values were presented for continuous variables, while frequencies and percentages were reported for categorical variables. Univariable and multivariable binary logistic regression analyses were performed to examine associations with DR/DME (the latter two variables were treated as dependent variables in the respective models); odds ratios (ORs) and 95% confidence intervals (CIs) were estimated. Variables that were proven significant at the univariable analysis were entered into the multivariable model.

In addition, multinomial logistic regression analysis was performed, with the NPDR and PDR status set as the dependent variable; the “no DR” group was set as the reference category of the model. The associations of variables with the other groups (NPDR and PDR) were reported as relative risks (RRs) and 95% confidence intervals (95% CIs). Once again, only the variables that were proven significant at the univariable analysis were incorporated into the multivariable multinomial logistic regression model [20,21].

The level of statistical significance was set at 0.05. Statistical analysis was performed using STATA/SE 16.0 statistical software (Stata Corporation, College Station, TX, USA).

## 3. Results

Of the initial cohort of 215 patients, 12 were excluded since they were out of age range for “ASCVD Risk Estimator Plus” calculator use. In addition, fifteen patients had a previous stroke, and seven had a previous myocardial infarction. As a result, participants in the analysis were 181 patients. Table 1 shows the demographic and clinical characteristics of the study sample. The mean age of patients was 67.0 years (SD 8.2) (median: 68 years). A total of 96 out of 181 patients (53.0%) were male and 85 (47%) were female. The mean duration of DM was 17.0 years (SD 10.7) (median: 17 years), and the mean HbA1c was 7.1% (SD 1.2%), (median: 6.8%). Regarding comorbidities, 123 out of 181 patients (68%) had hypertension and 138 (76.2%) dyslipidemia. Of note, 105 out of 181 patients (58.0%) had DR, 44 (24.3%) neuropathy, 16 (8.8%) nephropathy, and 11 (6.1%) diabetic foot. ASCVD score was calculated as low in 5 out of 181 patients (2.8%), borderline in 4 (2.2%), intermediate in 39 (21.5%), and high in 133 patients (73.5%).

The results of the univariable and multivariable binary logistic regression analysis of factors potentially correlated with DR are depicted in Table 2. A duration of DM greater than 17 years was associated with increased odds of DR development (OR = 4.24, 95% CI: 2.25–7.97, *p* < 0.001). Levels of HbA1c higher than 7% were also associated with DR (OR = 4.63, 95% CI: 2.38–8.99, *p* < 0.001). Patients on insulin were found to have higher odds of DR development (OR = 4.16, 95% CI: 2.18–7.95, *p* < 0.001). Regarding other complications of DM, the presence of neuropathy was associated with an increased likelihood of DR (OR = 4.43, 95% CI: 1.92–10.23, *p* < 0.001). Age (*p* = 0.475), sex (*p* = 0.318), smoking (*p* = 0.081), treatment of DM with other medications than insulin (*p* > 0.05 for all medications), statin use (*p* = 0.146), presence of hypertension (*p* = 0.280), dyslipidemia (*p* = 0.281), and nephropathy (*p* = 0.704) were not significantly associated with DR. Diabetic foot presented a trend for association with DR but did not reach statistical significance (*p* = 0.051). Additionally, the ASCVD score, as it was calculated with the “ASVCD Risk Estimator Plus” tool, was not found to be significantly correlated with DR (*p* = 0.191). The same significant results were replicated in a univariable multinomial logistic regression analysis, which examined factors separately associated with NPDR and PDR, as shown in Table 3.

At the multivariable binary logistic regression analysis (Table 2), the univariable association with insulin use dissipated, whereas factors that were associated with DR independently were DM duration (multivariable OR = 3.16, 95% CI: 1.55–6.44, *p* = 0.002), HbA1c levels (multivariable OR = 2.94, 95% CI: 1.37–6.32, *p* = 0.006), and the presence of neuropathy (multivariable OR = 3.59, 95% CI: 1.43–9.05, *p* = 0.007). At the multivariable multinomial logistic regression analysis, NPDR development was associated with duration of DM (multivariable RR = 3.31, 95% CI: 1.57–6.97, *p* = 0.002), HbA1c levels (multivariable RR = 2.24, 95% CI: 1.00–5.02, *p* = 0.050), and neuropathy (multivariable RR: 3.94, 95% CI: 1.54–10.11, *p* = 0.004), while PDR development was only associated with HbA1c levels (multivariable RR = 6.88, 95% CI: 2.19–21.63, *p* = 0.001).

Table 4 illustrates the results of the univariable and multivariable binary analysis of potential factors associated with DME. The male sex was found to be associated with DME (OR = 1.95, 95% CI: 1.02–3.73, *p* = 0.044), as well as duration of DM greater than 17 years (OR = 2.05, 95% CI: 1.08–3.92, *p* = 0.029), insulin use (OR = 2.38, 95% CI: 1.25–4.52, *p* = 0.008), and neuropathy (OR = 2.34, 95% CI: 1.16–4.73, *p* = 0.018). However, at the multivariable approach, only the male sex reached statistical significance (multivariable OR = 2.08, 95% CI: 1.06–4.10, *p* = 0.034).

It is worth noting that in the cohort of 22 patients who have exhibited previous stroke or myocardial infarction, 19 patients (86.4%) had DR.

## 4. Discussion

The principal message of this study is that ASCVD risk, as it was calculated based on the “ASCVD Risk Estimator Plus” tool, was high or intermediate in 95% of patients, suggesting that patients with type 2 DM are susceptible to an ASCVD event. However, the ASCVD risk was not significantly associated with DR. On the other hand, a duration of DM of more than 17 years, HbA1c of more than 7%, and neuropathy increase the odds of DR development by about three times. It is worth mentioning that PDR was only associated with HbA1c levels, underlining the importance of glycemic control.

It is widely known that ASCVD and DR share common risk factors, such as hypertension, dyslipidemia, and glycemic control [22]. Therefore, although several studies have shown a correlation between DR and ASCVD [12,23,24,25,26], there are other reports that have found that this association diminishes following correction for established ASCVD risk factors [13]. According to Eid et al., patients with type 2 DM and DR are a particularly high-risk group for CVD, and they need a customized treatment plan targeting lowering CVD risk factors, improving metabolic control, and monitoring CVD over time [23]. In addition, patients with advanced stages of DR appear to have a higher risk of CVD [7,23,27]. In our study, out of 22 patients who have exhibited stroke or myocardial infarction, 19 patients (86.4%) had DR, pointing out DR as a potential marker for CVD development.

However, in our study, the ASCVD risk based on the “ASCVD Risk Estimator Plus” was not associated with DR, although the ASCVD risk was found to be high in 73.5% and intermediate in 21.5% of patients with type 2 DM. This could be attributed to the use of this tool per se for the calculation of ASCVD risk. In fact, the 2019 European Society of Cardiology/European Association for the Study of Diabetes (ESC/EASD) joint guidelines on diabetes, pre-diabetes, and cardiovascular diseases do not recommend assessing ASCVD risk in patients with type 2 DM based on global risk scores derived in the general population [28]. By contrast, both the American Diabetes Association (ADA) and the American College of Cardiology/American Heart Association (ACC/AHA) clinical practice guidelines on the management of blood cholesterol in people with diabetes recommend a global estimation of ASCVD risk in patients with type 2 DM, using the “ASCVD Risk Estimator Plus” tool [29,30], as we used in this study. Noticeably, the “ASCVD Risk Estimator Plus” includes DM as a risk factor, but it does not account for type of DM (type 1 or 2), DM duration, or the presence of DM complications. In addition, Dziopa et al. in a comparison of 22 risk scores in patients with type 2 DM concluded that CVD risk prediction scores could not accurately identify individuals who experienced a CVD event in the 10 years of follow-up, while CVD risk scores derived in the general population performed worse in patients with type 2 DM, emphasizing the difficulties of accurately predicting CVD in a relatively high-risk population [31]. Therefore, we recommend that patients with DM type 2 should have a thorough cardiovascular examination for ASCVD risk assessment, and should not be based on ASCVD risk score calculators, which were constructed for the general population [32]. Finally, the fact that no association was found between DR and ASCVD risk score may suggest that other characteristics of the mechanistic pathways exist for retinal microvascular damage, enhancing the risk for DR progression in patients with diabetes, which might not be reflected by the shared CVD risk factors [18].

Regarding other factors, which were found to be associated with DR in this study, we confirmed the well-known association between duration of DM, as well as HbA1c levels, with DR development [33,34]. Interestingly, PDR was only associated with HbA1c levels, stressing the high importance of good glycemic control in the prevention of advanced DM complications [35]. Furthermore, the association between DR and diabetic neuropathy has also been described, since the two microvascular complications share common pathophysiological pathways, including the polyol pathway flux, the formation of advanced glycation end-products, the release of cytokines, the activation of protein kinase C, and oxidative stress [36].

A potential limitation of the study is the cross-sectional design. In addition, the use of the “ASCVD Risk Estimator Plus” as a tool for the assessment of CVD risk in patients with type 2 raised controversy and is another limitation of the study. Noticeably, the study sample in this cohort was free of CVD events, while it is well known that patients who are diabetic often have comorbidities, and therefore, it should be taken into account in the interpretation of our results.

In conclusion, we found that there was not a significant association between the ASCVD risk score estimated with “ASCVD Risk Estimator Plus” and DR in patients with type 2 DM, although 95% of patients had high or intermediate ASCVD risk scores. This finding cannot exclude an association between CVD and DR. The latter assumption could be supported by the fact that in 22 patients with stroke or myocardial infarction, about 87% had DR, suggesting that DR could be hypothesized to be associated with CVD events. It is worth noting that suitable tools for the estimation of ASCVD risk should be used in patients with DM so as to identify vulnerable population groups with high ASCVD risk and target these groups for treatment. Further prospective studies with a large sample size evaluating ASCVD risk based on thorough cardiological examination are needed to establish if DR could serve as a potential predictor for ASCVD risk in patients with type 2 DM.

## Figures and Tables

**Table 1:** Demographic and clinical characteristics of the study sample (n = 181 patients).

Age (Years, Mean (SD))	67.0 (SD 8.2)
Sex (n, %)	
*Male*	96 (53.0%)
*Female*	85 (47.0%)
Smoking (n, %)	
*Yes*	54 (29.8%)
*No*	127 (71.2%)
DM duration (years, mean (SD))	17.0 (SD 10.7)
HbA1c (%, mean (SD))	7.1 (SD 1.2)
Hypertension (n, %)	123 (68.0%)
Dyslipidemia (n, %)	138 (76.2%)
Diabetic retinopathy (n, %)	
*No*	76 (42.0%)
*Mild non-proliferative*	22 (12.2%)
*Moderate non-proliferative*	22 (12.2%)
*Severe non-proliferative*	33 (18.2%)
*Proliferative*	28 (15.4%)
Diabetic macular edema (n, %)	56 (30.9%)
Neuropathy (n, %)	44 (24.3%)
Nephropathy (n, %)	16 (8.8%)
Diabetic foot (n, %)	11 (6.1%)
Atherosclerotic cardiovascular disease score (n, %)	
*Low*	5 (2.8%)
*Borderline*	4 (2.2%)
*Intermediate*	39 (21.5%)
*High*	133 (73.5%)

DM: diabetes mellitus; SD: standard deviation.

**Table 2:** Univariable and multivariable binary logistic regression analysis of factors associated with diabetic retinopathy.

	DR (n, %)	Univariable OR (95% CI)	*p*	Multivariable OR (95% CI)	*p*
**Age**					
*<65 years*	32/59 (54.2)	Ref.			
*≥65 years*	73/122 (59.8)	1.26 (0.67–2.35)	0.475		
**Sex**					
*Male*	59/96 (61.5)	1.35 (0.75–2.44)	0.318		
*Female*	46/85 (54.1)	Ref.			
**Smoking**					
*Yes*	26/54 (48.2)	0.56 (0.30–1.07)	0.081		
*No*	79/127 (62.2)	Ref.			
**Diabetes mellitus** **duration**					
*<17 years* *(median)*	37/90 (41.1)	Ref.		Ref.	
*≥17 years*	68/91 (74.7)	4.24 (2.25–7.97)	**<0.001**	3.16 (1.55–6.44)	**0.002**
**HbA1c**					
*<7%*	45/104 (43.3)	Ref.		Ref.	
*≥7%*	60/77 (77.9)	4.63 (2.38–8.99)	**<0.001**	2.94 (1.37–6.32)	**0.006**
**Treatment of DM**					
***Insulin***				Ref.	
*Yes*	61/80 (76.3)	4.16 (2.18–7.95)	**<0.001**	1.54 (0.70–3.38)	0.286
*No*	44/101 (43.6)	Ref.			
***Metformin***					
*Yes*	74/134 (55.2)	0.64 (0.32–1.27)	0.201		
*No*	31/47 (66.0)	Ref.			
***Glucagon-like peptide-1*** ***(G*** ***LP-1)***					
*Yes*	29/51 (56.9)	0.94 (0.49–1.80)	0.845		
*No*	76/130 (58.5)	Ref.			
***Sodium–glucose co-transporters 2 inhibitors***					
*Yes*	48/76 (63.2)	1.44 (0.79–2.64)	0.233		
*No*	57/105 (54.3)	Ref.			
***Dipeptidyl peptidase-*** ***4 inhibitors***					
*Yes*	35/59 (59.3)	1.08 (0.58–2.04)	0.804		
*No*	70/122 (57.4)	Ref.			
***Sulfonylurea***					
*Yes*	12/20 (60.0)	1.10 (0.43–2.83)	0.848		
*No*	93/161 (57.8)	Ref.			
***Thiazolidine***					
*Yes*	8/10 (80.0)	3.05 (0.63–14.80)	0.166		
*No*	97/171 (56.7)	Ref.			
**Hypertension**					
*Yes*	68/123 (55.3)	0.70 (0.37–1.33)	0.280		
*No*	37/58 (63.8)	Ref.			
**Dyslipidemia**					
*Yes*	77/138 (55.8)	0.68 (0.33–1.38)	0.281		
*No*	28/43 (65.1)	Ref.			
**Statin use**					
*Yes*	79/143 (55.2)	0.57 (0.27–1.22)	0.146		
*No*	26/38 (68.4)	Ref.			
**Neuropathy**					
*Yes*	36/44 (81.8)	4.43 (1.92–10.23)	**<0.001**	3.59 (1.43–9.05)	**0.007**
*No*	69/137 (50.4)	Ref.		Ref.	
**Nephropathy**					
*Yes*	10/16 (62.5)	1.23 (0.43–3.54)	0.704		
*No*	95/165 (57.6)	Ref.			
**Diabetic foot**					
*Yes*	10/11 (90.9)	7.89 (0.99–63.05)	0.051		
*No*	95/170 (55.9)	Ref.			
**Aspirin use**					
*Yes*	26/51 (51.0)	0.67 (0.35–1.29)	0.231		
*No*	79/130 (60.8)	Ref.			
**ASCVD** **score**					
*Low/* *b* *orderline/* *i* *ntermediate*	24/48 (50.0)	Ref.		Ref.	
*High*	81/133 (60.9)	1.56 (0.80–3.03)	0.191		

ASCVD: atherosclerotic cardiovascular disease; CI: confidence interval; DM: diabetic mellitus; DR: diabetic retinopathy; OR: odds ratio; Ref.: reference.

**Table 3:** Univariable and multivariable multinomial logistic regression analyses of factors associated with non-proliferative and proliferative diabetic retinopathy, separately.

		NPDR Versus No DR Comparison					PDR Versus No DR Comparison			
	NPDR (n, %)	Univariable RR (95% CI)	*p*	Multivariable RR (95% CI)	*p*	PDR (n, %)	Univariable RR (95% CI)	*p*	Multivariable RR (95% CI)	*p*
**Age**										
*<65 years*	18/59 (30.5)	Ref.		Ref.		14/59 (23.7)	Ref.		Ref.	
*≥65 years*	59/122 (48.4)	1.81 (0.89–3.66)	0.101			14/122 (11.5)	0.55 (0.23–1.32)	0.183		
**Sex**										
*Male*	41/96 (42.7)	1.20 (0.64–2.26)	0.573			18/96 (18.8)	1.90 (0.78–4.64)	0.160		
*Female*	36/85 (42.4)	Ref.				10/85 (11.8)	Ref.			
**Smoking**										
*Yes*	19/54 (35.2)	0.56 (0.28–1.13)	0.105			7/54 (13.0)	0.57 (0.22–1.51)	0.260		
*No*	58/127 (45.7)	Ref.				21/127 (16.5)	Ref.			
**DM duration**										
<17 years	28/90 (31.7)	Ref.				9/90 (10.0)				
≥17 years	49/91 (53.9)	4.03 (2.05–7.92)	**<0.001**	3.31 (1.57–6.97)	**0.002**	19/91 (20.9)	4.86 (1.92–12.35)	**0.001**	2.74 (0.96–7.82)	0.059
**HbA1c**										
<7%	39/104 (37.5)	Ref.				6/104 (5.8)	Ref.			
≥7%	38/77 (49.4)	3.38 (1.68–6.81)	**0.001**	2.24 (1.00–5.02)	**0.050**	22/77 (28.6)	12.73 (4.45–36.42)	**<0.001**	6.88 (2.19–21.63)	**0.001**
**Treatment of DM**										
***Insulin***										
*Yes*	40/80 (50.0)	3.24 (1.63–6.43)	**0.001**	1.29 (0.56–2.96)	0.545	21/80 (26.3)	9.00 (3.31–24.48)	**<0.001**	2.72 (0.85–8.70)	0.091
*No*	37/101 (36.6)	Ref.				7/101 (6.9)	Ref.			
***Metformin***										
*Yes*	56/134 (41.8)	0.71 (0.34–1.50)	0.370			18/134 (13.4)	0.48 (0.19–1.24)	0.130		
*No*	21/47 (44.7)	Ref.				10/47 (21.3)	Ref.			
***GLP-1***										
*Yes*	23/51 (45.1)	1.05 (1.52–2.10)	0.900			6/51 (11.8)	0.67 (0.24–1.87)	0.445		
*No*	54/130 (41.5)	Ref				22/130 (16.9)	Ref.			
***SGLT-2***										
*Yes*	33/76 (43.4)	1.29 (0.67–2.46)	0.448			15/76 (19.7)	1.98 (0.82–4.75)	0.127		
*No*	44/105 (41.9)	Ref.				13/105 (12.4)	Ref.			
***DPP-4***										
*Yes*	30/59 (50.9)	1.38 (0.71–2.69)	0.340			5/59 (8.5)	0.47 (0.16–1.39)	0.172		
*No*	47/122 (38.5)	Ref.				23/122 (18.9)	Ref.			
***Sulfonylurea***										
*Yes*	9/20 (45.0)	1.13 (0.41–3.09)	0.819			3/20 (15.0)	1.02 (0.25–4.15)	0.978		
*No*	68/161 (42.2)	Ref.				25/161 (15.5)	Ref.			
***Thiazolidine***										
*Yes*	7/10 (70.0)	3.70 (0.74–18.42)	0.110			1/10 (10.0)	1.37 (0.12–15.73)	0.800		
*No*	70/171 (40.9)	Ref.				27/171 (15.8)	Ref.			
**Hypertension**										
*Yes*	52/123 (42.3)	0.79 (0.40–1.59)	0.515			16/123 (13.0)	0.51 (0.21–1.25)	0.142		
*No*	25/58 (43.1)	Ref.				12/58 (20.7)	Ref.			
**Dyslipidemia**										
*Yes*	56/128 (40.6)	0.66 (0.31–1.40)	0.274			21/138 (15.2)	0.74 (0.26–2.06)	0.561		
*No*	21/43 (48.8)	Ref.				7/43 (16.3)	Ref.			
**Statin use**										
*Yes*	57/143 (39.9)	0.53 (0.24–1.19)	0.125			22/143 (15.4)	0.69 (0.23–2.05)	0.502		
*No*	20/38 (52.6)	Ref.				6/38 (15.8)	Ref.			
**Neuropathy**										
*Yes*	27/44 (61.4)	4.59 (1.92–10.95)	**0.001**	3.94 (1.54–10.11)	**0.004**	9/44 (20.5)	4.03 (1.37–11.85)	**0.011**	2.51 (0.75–8.42)	0.137
*No*	50/137 (36.5)	Ref.				19/137 (13.9)	Ref.			
**Nephropathy**										
*Yes*	5/16 (31.3)	0.81 (0.24–2.78)	0.738			5/16 (31.3)	2.54 (0.71–9.09)	0.153		
*No*	72/165 (43.6)	Ref.				23/165 (13.9)	Ref.			
**Diabetic foot**										
*Yes*	7/11 (63.6)	7.5 (0.90–62.51)	0.063			3/11 (27.3)	9.00 (0.90–90.49)	0.062		
*No*	70/170 (41.2)	Ref.				25/170 (14.7)	Ref.			
**Aspirin use**										
*Yes*	19/51 (37.3)	0.67 (0.33–1.35)	0.263			7/51 (13/7)	0.68 (0.26–1.81)	0.441		
*No*	58/130 (44.6)	Ref.				21/130 (16.2)	Ref.			
**ASCVD**										
*Low/borderline/* *intermediate*	17/48 (35.4)	Ref.				7/48 (14.6)	Ref.			
*High*	60/133 (45.1)	1.63 (0.79–3.36)	0.186			21/133 (15.8)	1.38 (0.52–3.70)	0.516		

ASCVD: atherosclerotic cardiovascular disease; CI: confidence interval; DM: diabetic mellitus; DR: diabetic retinopathy; DPP-4: dipeptidyl peptidase-4 inhibitors; GLP-1: glucagon-like peptide-1; NPDR: non-proliferative diabetic retinopathy; PDR: proliferative diabetic retinopathy; Ref.: reference; RR: relative risk; SGLT-2: sodium–glucose co-transporters 2 inhibitors.

**Table 4:** Univariable and multivariable binary logistic regression analysis of factors associated with diabetic macular edema (DME).

	DΜΕ (n, %)	Univariable OR (95% CI)	*p*	Multivariable OR (95% CI)	*p*
**Age**					
*<65 years*	16/59 (27.1)	Ref.			
*≥65 years*	40/122 (32.8)	1.31 (0.66–2.61)	0.440		
**Sex**					
*Male*	36/96 (37.5)	1.95 (1.02–3.73)	**0.044**	2.08 (1.06–4.10)	**0.034**
*Female*	20/85 (23.5)	Ref.		Ref.	
**Smoking**					
*Yes*	15/55 (27.8)	0.81 (0.40–1.63)	0.549		
*No*	41/127 (32.3)	Ref.			
**DM duration**					
<17 years	21/90 (23.3)	Ref.		Ref.	
≥17 years	35/91 (38.5)	2.05 (1.08–3.92)	**0.029**	1.69 (0.83–3.44)	0.147
**HbA1c**					
<7%	29/104 (27.9)	Ref.			
≥7%	27/77 (35.1)	1.40 (0.74–2.63)	0.302		
**Treatment of DM**					
***Insulin***					
*Yes*	33/80 (41.3)	2.38 (1.25–4.52)	**0.008**	1.83 (0.89–3.76)	0.102
*No*	23/101 (22.8)	Ref.			
***Metformin***					
*Yes*	43/134 (32.1)	1.24 (0.59–2.58)	0.572		
*No*	13/47 (27.7)	Ref.			
***GLP-1***					
*Yes*	19/51 (37.3)	1.49 (0.75–2.96)	0.251		
*No*	37/130 (28.5)	Ref.			
***SGLT-2***					
*Yes*	27/76 (35.5)	1.44 (0.76–2.73)	0.257		
*No*	29/105 (27.6)	Ref.			
***DPP-4***					
*Yes*	18/59 (30.5)	0.97 (0.49–1.90)	0.931		
*No*	38/122 (31.2)	Ref.			
***Sulfonylurea***					
*Yes*	9/20 (45.0)	1.98 (0.77–5.10)	0.155		
*No*	47/161 (29.2)	Ref.			
***Thiazolidine***					
*Yes*	6/10 (60.0)	3.63 (0.98–13.42)	0.053		
*No*	50/171 (29.2)	Ref.			
**Hypertension**					
*Yes*	35/123 (28.5)	0.70 (0.36–1.36)	0.293		
*No*	21/58 (36.2)	Ref.			
**Dyslipidemia**					
*Yes*	41/138 (29.7)	0.79 (0.38–1.63)	0.522		
*No*	15/43 (34.9)	Ref.			
**Statin use**					
*Yes*	41/143 (28.7)	0.62 (0.29–1.30)	0.203		
*No*	15/38 (39.5)	Ref.			
**Neuropathy**					
*Yes*	20/44 (45.5)	2.34 (1.16–4.73)	**0.018**	1.83 (0.87–3.86)	0.112
*No*	37/137 (26.3)	Ref.		Ref.	
**Nephropathy**					
*Yes*	6/16 (37.5)	1.38 (0.48–4.00)	0.553		
*No*	50/165 (30.3)	Ref.			
**Diabetic foot**					
*Yes*	6/11 (54.6)	2.88 (0.84–9.87)	0.092		
*No*	50/170 (29.4)	Ref.			
**Aspirin use**					
*Yes*	14/51 (27.5)	0.79 (0.39–1.62)	0.525		
*No*	42/130 (32.3)	Ref.			
**ASCVD score**					
*Low/* *b* *orderline/* *i* *ntermediate*	11/48 (22.9)	Ref.		Ref.	
*High*	45/133 (33.8)	1.72 (0.80–3.69)	0.164		

ASCVD: atherosclerotic cardiovascular disease; CI: confidence interval; DM: diabetic mellitus; DME: diabetic macular edema; DPP-4: dipeptidyl peptidase-4 inhibitors; GLP-1: glucagon-like peptide-1; OR: odds ratio; Ref.: reference; SGLT-2: sodium–glucose co-transporters 2 inhibitors.

## Data Availability

Data are available upon request.
